# Localized and Controlled Delivery of Nitric Oxide to the Conventional Outflow Pathway via Enzyme Biocatalysis: Toward Therapy for Glaucoma

**DOI:** 10.1002/adma.201604932

**Published:** 2017-02-21

**Authors:** Rona Chandrawati, Jason Y. H. Chang, Ester Reina‐Torres, Coline Jumeaux, Joseph M. Sherwood, W. Daniel Stamer, Alexander N. Zelikin, Darryl R. Overby, Molly M. Stevens

**Affiliations:** ^1^ Department of Materials Department of Bioengineering and Institute of Biomedical Engineering Imperial College London London SW7 2AZ UK; ^2^ Department of Bioengineering Imperial College London London SW7 2AZ UK; ^3^ Department of Ophthalmology Duke University School of Medicine Durham NC 27710 USA; ^4^ Department of Chemistry and iNANO Interdisciplinary Nanoscience Center Aarhus University Aarhus C 8000 Denmark

**Keywords:** β‐galactosidase, glaucoma, layer‐by‐layer capsules, liposomes, nitric oxide

## Abstract

**Nitric oxide (NO)** is able to lower intraocular pressure (IOP); however, its therapeutic effects on outflow physiology are location‐ and dose‐dependent. A NO delivery platform that directly targets the resistance‐generating region of the conventional outflow pathway and locally liberates a controlled dose of NO is reported. An increase in outflow facility (decrease in IOP) is demonstrated in a mouse model.

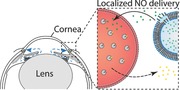

Glaucoma is the second leading cause of irreversible blindness that affects more than 60 million people worldwide. Elevated intraocular pressure (IOP) is the primary risk factor,^1^, ^2^ and reducing IOP is the only clinical approach to prevent further glaucomatous vision loss.^3^, ^4^ All daily therapies, however, fail to achieve sufficient IOP reduction, likely because they do not target the conventional outflow pathway that controls IOP and becomes diseased in glaucoma.^2^ The conventional outflow pathway is the primary route of aqueous humor outflow from the eye, and increased resistance deep in this pathway, where the trabecular meshwork (TM) and Schlemm's canal (SC) inner wall interact, causes IOP elevation in glaucoma. Nitric oxide (NO), a key signaling molecule responsible for mediating a wide array of physiological roles across multiple biological systems, decreases outflow resistance and lowers IOP in various animal models and human patients.^5^, ^6^, ^7^, ^8^, ^9^, ^10^, ^11^, ^12^, ^13^ Due to the therapeutic potential of NO, many efforts have focused on developing NO‐donating compounds for glaucoma. In particular, latanoprostene bunod (VESNEO; Bausch and Lomb), a modified version of the existing glaucoma drug latanoprost, contains an NO‐donating moiety and is modestly effective at reducing elevated IOP by ≈1.5 mmHg over latanoprost alone in ocular hypertensive patients.^11^, ^12^, ^13^ Despite the promise of NO as a glaucoma therapy, significant challenges remain before the full IOP‐reducing potential of NO can be realized.

NO plays a multifaceted role within the conventional outflow pathway such that its therapeutic effect depends strongly upon the location and concentration of NO delivered.^14^ Some effects of NO may also be counterproductive and tend to cause IOP elevation that opposes the desired therapeutic outcome. For instance, NO delivery to the smooth muscle cells of the ciliary muscle (CM) causes CM relaxation.^15^ As CM tension maintains patency of the conventional outflow pathway, NO‐induced relaxation of the CM increases outflow resistance and elevates IOP.^15^, ^16^, ^17^ Acting as a vasodilator, NO may also increase IOP by increasing downstream episcleral venous pressure^18^ or by altering choroidal blood volume.^19^ Dosing is also important, as high levels of NO can exacerbate off‐target effects.^20^ Furthermore, the reactive nature of NO and its short half‐life only allows NO to diffuse over short distances,^21^, ^22^ which severely limits the efficacy of topical drug preparations from reaching deep into the outflow pathway where resistance is generated. In order to fully exploit the IOP‐reducing potential of NO, it is therefore necessary to achieve local and controlled delivery of NO deep in the conventional outflow pathway. To our knowledge, NO‐mediated IOP‐lowering therapeutics that primarily target the conventional outflow pathway do not currently exist.

Here, we develop and test an NO delivery platform that directly targets the conventional outflow pathway and locally liberates a controlled dose of NO via enzyme biocatalysis (**Scheme** **1**a). In our approach, enzymes are embedded at the desired sites and serve as biological machinery that can locally convert externally administered NO donors into active therapeutics. To enmesh enzymes deep within the TM, which is the principal resistance‐generating region, we encapsulate β‐galactosidase in polymer carriers. We fabricate polymer carrier capsules via layer‐by‐layer adsorption of interacting polymers onto sacrificial particle templates,^23^, ^24^, ^25^, ^26^, ^27^, ^28^ a versatile technique that allows incorporation of an extensive choice of materials within the multilayer structures and gives fine control over the diffusion of molecules across the shell of the polymer capsules. In this study, we chose hydrogen‐bonded polymer pairs based on thiol‐functionalized poly(methacrylic acid) (PMA_SH_) and poly(*N*‐vinylpyrrolidone) (PVP) as the system to encapsulate enzyme β‐galactosidase (Scheme 1b) due to their high colloidal stability, (bio)degradability, and biocompatibility.^29^, ^30^, ^31^ These capsules have previously been used to encapsulate a range of functional and active biomolecules, including enzymes,^32^, ^33^, ^34^ DNA,^35^, ^36^ anticancer drugs,^37^, ^38^ antigenic peptides,^30^, ^39^ and intact proteins.^31^, ^40^ Successful delivery of vaccines using PMA carriers have been demonstrated in vivo.^31^ We encapsulate NO donor, β‐gal‐NONOate, in liposomes because these lipid nanocarriers allow for sustained release of biomolecules and their properties can be modulated through the judicious choice of the lipid composition.^41^, ^42^, ^43^ Liposomes have been used to encapsulate latanoprost^44^ and plasmid DNA,^45^ and injected into eyes in vivo. In our study, liposomes are delivered to the outflow pathway. Upon degradation of the lipid vesicles, NO donors are slowly released to the TM, where the enzyme is enmeshed, and enzymatic activity of β‐galactosidase results in local delivery of active therapeutic NO at the outflow resistance sites, thus achieving an on‐site NO delivery to the conventional outflow pathway.

**Scheme 1:**
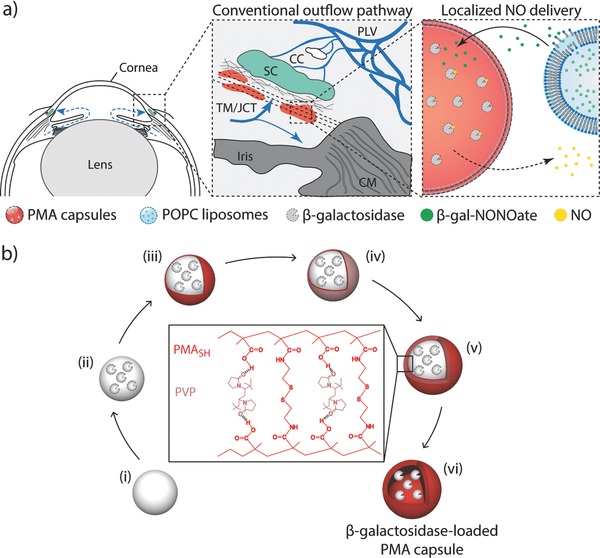
a) Schematic illustration of localized delivery of nitric oxide (NO) to the conventional outflow pathway via enzyme biocatalysis. Left: Anterior segment of eye showing the direction of aqueous humor flow in blue. Center: Enlargement of the iridocorneal angle (boxed region in left panel) showing the conventional outflow pathway. Right: Schematic of localized NO delivery within the trabecular meshwork (TM) near Schlemm's canal (SC). β‐Galactosidase is encapsulated in poly(methacrylic acid) (PMA) capsules and enmeshed within the TM. Liposomes containing NO donors (β‐gal‐NONOate) are delivered to the outflow pathway. Upon liposome degradation, NO donors are slowly released at the outflow resistance sites and enzymatic activity of β‐galactosidase results in local delivery of active therapeutic NO at the outflow resistance sites, achieving a targeted on‐site NO delivery to the conventional outflow pathway. CC: collector channels, CM: ciliary muscle, JCT: juxtacanalicular connective tissue, and PLV: perilimbal vessels. b) Schematic illustration of assembly of β‐galactosidase‐loaded PMA capsules via layer‐by‐layer technique. i) Aminated silica particle template is coated with ii) β‐galactosidase, followed by sequential deposition of iii) thiol‐functionalized PMA (PMA_SH_) and iv) poly(*N*‐vinylpyrrolidone) (PVP) via hydrogen bonding. v) Once four bilayers of PMA_SH_/PVP are deposited, the thiol groups of PMA_SH_ are oxidized into bridging disulfide linkages. vi) Removal of the sacrificial particle template results in (bio)degradable disulfide‐cross‐linked β‐galactosidase‐loaded PMA capsule.

In the current study, we: i) assemble β‐galactosidase‐loaded polymer capsules and β‐gal‐NONOate‐loaded liposomes, ii) demonstrate local release of NO via biocatalysis of β‐gal‐NONOate by β‐galactosidase and investigate the reaction kinetics in vitro, iii) demonstrate control over the dose and time of NO release via an enzyme‐prodrug mechanism, and iv) demonstrate the effect of localized delivery of NO to the conventional outflow pathway on outflow resistance in mouse models. Here, we present direct evidence that on‐site delivery of NO to the outflow resistance sites provides efficacious therapeutic alteration of aqueous humor dynamics.

We first investigated NO release kinetics achieved via catalytic activity of β‐galactosidase on β‐gal‐NONOate in mock aqueous humor solution (DBG solution: Dulbecco's phosphate buffered saline with divalent cations and 5.5 × 10^−3^ m glucose). We used an NO‐sensitive electrode immersed in β‐galactosidase solution (0.1 mg mL^−1^) and measured changes in NO concentration over time in response to the addition of β‐gal‐NONOate (50 × 10^−6^ m). β‐Galactosidase catalyzes the release of NO by hydrolysis of the glycosidic bonds. Upon addition of NO donors into the β‐galactosidase solution, an increasing production of NO was detected, followed by a decline back to the original baseline signal (**Figure** **1**a). This reaction generated NO with an average half‐life (*t*_1/2_) of ≈5 min at physiological conditions (pH 7.4 and 37 °C), which refers to the time to reach 50% NO decay. With the same concentration of enzyme (0.1 mg mL^−1^ β‐galactosidase) and an increasing amount of added substrates, NO can be released in a dose‐dependent manner (Figure 1b). The concentration of NO generated by β‐galactosidase‐mediated hydrolysis of β‐gal‐NONOate was derived by correlation with a calibration curve (Figure S1, Supporting Information). The release of NO followed a 1:2 stoichiometry and exhibited a linear relationship. These β‐galactosidase (0.1 mg mL^−1^)/β‐gal‐NONOate (50 × 10^−6^ m) pairs released NO at physiologically relevant concentrations required to activate soluble guanylate cyclase in the TM to increase outflow facility and decrease elevated IOP in vivo.^8^, ^21^, ^46^, ^47^

**Figure 1:**
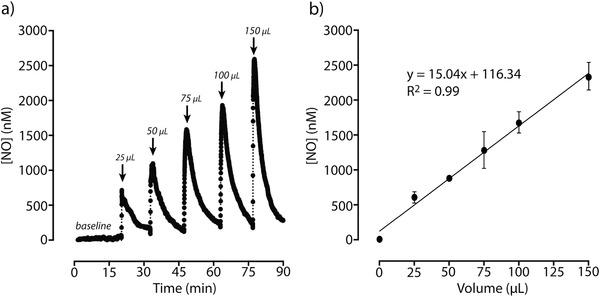
Nitric oxide (NO) release by β‐galactosidase‐mediated hydrolysis of β‐gal‐NONOate. a) Representative dose‐response curve of NO generated from enzymatic hydrolysis of increasing boluses of β‐gal‐NONOate (50 × 10^−6^ m) by β‐galactosidase (0.1 mg mL^−1^) in DBG solution (Dulbecco's PBS with divalent cations and 5.5 × 10^−3^ m glucose) at 37 °C. NO release was measured using an NO‐sensitive probe (ISO‐NO Mark II). b) Linear relationship between NO released and increasing volume of non‐encapsulated β‐gal‐NONOate when hydrolyzed by β‐galactosidase (*n* = 3; error bars are SD).

Using the same setup, we next investigated the kinetics of NO release when enzymes and NO donors were spatially separated and encapsulated in polymer capsules and liposomes, respectively. Here, β‐galactosidase was adsorbed onto amine‐functionalized silica particles, followed by the sequential deposition of four bilayers of PMA_SH_ and PVP. The thiol groups within the polymer layers were cross‐linked with 2,2′‐dithiodipyridine, and disulfide‐stabilized hollow PMA capsules containing β‐galactosidase were obtained upon dissolution of the silica templates (Scheme 1b). To confirm the encapsulation of β‐galactosidase, we conjugated the enzyme with Alexa Fluor 488 dye. Fluorescence microscopy imaging of capsules containing Alexa Fluor 488‐labeled β‐galactosidase showed a homogeneous green corona on the capsule membrane (Figure S2, Supporting Information). The hollow capsules were intact, non‐agglomerated, and preserved the spherical shape of the particle templates. The NO donor, β‐gal‐NONOate, was encased within zwitterionic 1‐palmitoyl‐2‐oleoyl‐*sn*‐glycero‐3‐phosphocholine (POPC) liposomes.

Here, an NO‐sensitive electrode was immersed and equilibrated in a suspension of capsules containing β‐galactosidase in DBG solution (10^6^ capsules µL^−1^), and we measured NO release in response to the addition of β‐gal‐NONOate‐loaded liposomes (50 × 10^−6^ m β‐gal‐NONOate, 10^6^ liposomes µL^−1^) within a glass vial. This reaction was performed at 37 °C on a hot plate with continuous mixing. Upon introduction of liposomes to the capsule suspension, a maximal NO concentration of ≈900 × 10^−9^ m was generated in 30 min, followed by a gradual decay over 6 h to the baseline signal (**Figure** **2**a). By spatial separation and encapsulation of enzymes and NO donors into polymer capsules and liposomes, respectively, the half‐life of NO released increased 30‐fold, extending the *t*_1/2_ from ≈5 min to ≈2.5 h, which is highly beneficial for treatment that requires sustained delivery of NO. NO donors were gradually released from lipid vesicles over time, freely permeating through the shell of the PMA capsules and were subsequently hydrolyzed by β‐galactosidase. This led to a staggered generation of NO greater than the depletion of NO in solution. Successful generation of NO indicated that core removal did not cause loss of enzyme activity in the capsules, as previously reported.^33^, ^34^, ^36^

**Figure 2:**
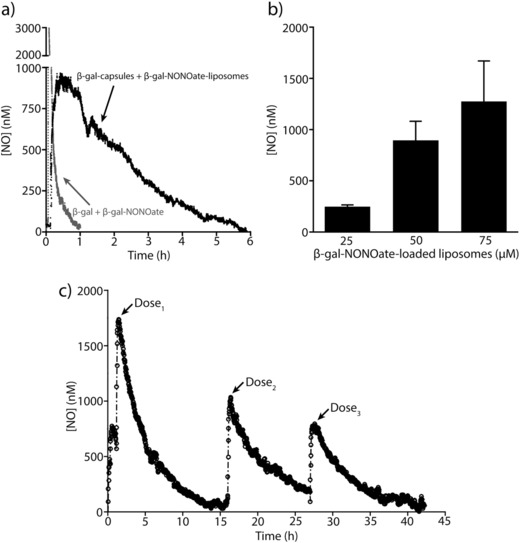
Nitric oxide (NO) release from β‐galactosidase encapsulated in capsules and β‐gal‐NONOate loaded in liposomes. a) Representative NO decay curve from enzymatic hydrolysis of β‐gal‐NONOate from liposomes (50 × 10^−6^ m, 10^6^ liposomes µL^−1^) by β‐galactosidase encapsulated in capsules (10^6^ capsules µL^−1^) or enzymatic hydrolysis of non‐encapsulated β‐gal‐NONOate and β‐galactosidase in DBG solution at 37 °C. By spatially separating enzymes and NO donors into polymer capsules and liposomes, respectively, the half‐life of NO released increased significantly, extending the *t*_1/2_ from ≈5 min to ≈2.5 h. A maximal NO concentration of ≈900 × 10^−9^ m was generated in 30 min. b) Maximal NO concentration generated in 30 min as a function of increasing loading concentration of β‐gal‐NONOate encapsulated inside the liposomes (25 × 10^−6^ to 75 × 10^−6^ m β‐gal‐NONOate, 10^6^ liposomes µL^−1^), which were added into a suspension of capsules containing β‐galactosidase (10^6^ capsules µL^−1^) in DBG solution at 37 °C (*n* = 3 for each concentration; error bars are SD). c) Sustained release of NO from β‐galactosidase encapsulated in PMA capsules and β‐gal‐NONOate loaded in liposomes. Representative NO decay curve from enzymatic hydrolysis of β‐gal‐NONOate from liposomes (75 × 10^−6^ m, 10^6^ liposomes µL^−1^) by β‐galactosidase encapsulated in capsules (10^6^ capsules µL^−1^) in DBG solution at 37 °C is shown. The enzymatic catalysis was repeated over three cycles by removing the hydrolyzed substrates and adding fresh β‐gal‐NONOate‐loaded liposomes into the suspension of capsules containing β‐galactosidase. Over 42 h the enzyme retained 50% of its activity.

To demonstrate the ability to control the dose of released NO, we spiked varying concentrations of β‐gal‐NONOate‐loaded liposomes (25 × 10^−6^ to 75 × 10^−6^ m β‐gal‐NONOate, 10^6^ liposomes µL^−1^) into the same suspension of capsules containing β‐galactosidase in DBG solution (10^6^ capsules µL^−1^). We observed a dose‐dependent generation of NO (250 × 10^−9^ to 1250 × 10^−9^ m, which corresponded to the maximal NO concentration generated in 30 min) as a function of increasing concentration of β‐gal‐NONOate encapsulated inside the liposomes (Figure 2b). These data demonstrated that through enzyme biocatalysis, the desired amount of NO delivery can be tuned independently via the choice of concentration of administered NO donors.

To demonstrate a sustained delivery of NO, we repeated the enzymatic catalysis over three cycles by removing the hydrolyzed substrates and adding fresh β‐gal‐NONOate‐loaded liposomes (75 × 10^−6^ m β‐gal‐NONOate, 10^6^ liposomes µL^−1^) into the suspension of capsules containing β‐galactosidase in DBG solution (10^6^ capsules µL^−1^). Using methodology used in this work, over 42 h, the enzyme retained at least 50% of its activity (Figure 2c), and this approach allows NO delivery that can be tuned at the desired units of time through the rate of liposome administration. We are now investigating means to extend the lifetime of the enzyme and to provide for greater duration of treatment.

To simulate the effects of localized delivery of NO in vivo and to evaluate their impact on a physiological parameter (conventional outflow), we measured outflow facility (*C*_r_) in enucleated eyes from C57BL/6 male wild‐type mice. Outflow facility is the mathematical inverse of outflow resistance. This study consists of a two‐step experimental protocol: i) delivery of β‐galactosidase‐loaded capsules (1.5 × 10^6^ capsules) to conventional outflow tissues via intracameral injections in living mice, and ii) ex vivo delivery of β‐gal‐NONOate‐loaded liposomes (10^6^ liposomes) during perfusion of enucleated eyes 48 h after intracameral injections. This approach allowed for sufficient recovery time and for the capsules to be enmeshed into active filtration regions of the TM prior to administration of β‐gal‐NONOate‐loaded liposomes. Microparticles delivered into the TM may induce IOP elevations in otherwise healthy mice.^48^, ^49^ However, this response typically requires larger particles (e.g., 15 µm^48^) or the addition of 10 mg mL^−1^ hyaluronate solution in addition to smaller particles (1–6 µm^49^) similar in size to those used in the current study. To control for potential IOP elevation, sham‐treated eyes were injected with otherwise identical capsules lacking enzyme. The use of enucleated eyes allowed for accurate measurements of outflow facility in mice with the iPerfusion system^50^ by eliminating pressure‐independent outflow, aqueous humor inflow, and episcleral venous pressure, which are only present in living mice. Previous studies have shown that ex vivo eyes remain pharmacologically responsive to NO for up to several hours^6^ and respond to receptor mediated compounds for up to 24 h.^51^

All experiments used paired eyes, where the experimental eye received capsules containing β‐galactosidase, while the contralateral control eye received empty capsules. Both eyes were enucleated for ex vivo mouse eye perfusions, where the perfusate consisted of liposomes containing β‐gal‐NONOate (50 × 10^−6^ m) in DBG solution, and were pressurized at 8 mmHg to allow the eyes to acclimatize to the pressure and temperature environment. After the acclimatization period, eyes were perfused over seven increasing pressure steps, from 6 to 15 mmHg. **Figure** **3**a shows a sample flow‐pressure plot comparing contralateral control and treated eyes, which indicates an apparent increasing effect of NO delivery on the flow rate. To determine the effect of localized NO delivery on *C*_r_, pressure and flow data for each step were averaged, and a power law regression was fit to the data according to *Q* = *C*_r_(*P/P*_r_)*^β^P*, where *Q* is the flow rate, *P* is the pressure, *P*_r_ is the reference pressure, *C*_r_ is the reference facility at *P*_r_, and β characterizes the non‐linearity of the data (see the Experimental Section for a description of outflow facility analysis). The delivery of β‐gal‐NONOate‐loaded liposomes in the presence of capsules containing β‐galactosidase resulted in an average increase in *C*_r_ by 84% [23, 177%] (mean [95% CI]) relative to vehicle‐treated contralateral eyes (*p* = 0.011, *n* = 7 pairs, weighted *t*‐test, average facility values: 6.50*^x^*/1.57 nL min^−1^ mmHg^−1^ vs 3.55*^x^*/1.38 nL min^−1^ mmHg^−1^, Figure 3b; see the Experimental Section for a description of statistical reporting).

**Figure 3:**
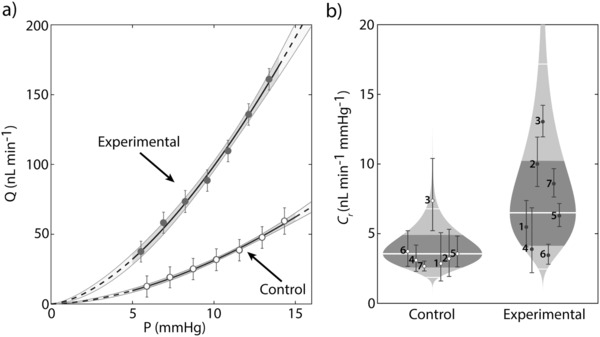
Effects of localized delivery of nitric oxide (NO) on conventional outflow facility in eyes obtained from C57BL/6 mice. Paired eyes were injected with either capsules containing β‐galactosidase (experimental) or empty capsules (control) 48 h prior to ex vivo mouse eye perfusions. Enucleated eyes were perfused with β‐gal‐NONOate‐loaded liposomes (50 × 10^−6^ m) to determine the flow‐pressure relationships for paired eyes. a) Representative flow‐pressure relationship for a given pair of eyes is shown. Solid lines represent the power law fitting of the data set. Shaded regions represent the 95% confidence bounds on the fitting. Error bars represent the two standard deviations for the flow rate at each pressure step. b) Cello plots showing relative difference in outflow facility (*C*_r_) between contralateral eyes perfused with β‐gal‐NONOate‐loaded liposomes (*p* = 0.011, *n* = 7, weighted *t*‐test). The delivery of β‐gal‐NONOate‐loaded liposomes in the presence of capsules containing β‐galactosidase resulted in an average increase in *C*_r_ by 84% [23, 177%] relative to vehicle‐treated contralateral eyes. The shaded regions represent the log‐normal distribution that best describes the data, while the thick white central lines indicate the geometric means. Error bars represent the 95% confidence intervals on the relative difference in facility. The dark shaded regions indicate the 95% confidence intervals on the geometric mean, and the outer thin white lines indicate the range that approximately encompasses 95% of the measured data.

In the current study, the structural integrity of the β‐galactosidase‐loaded PMA capsules allowed them to be localized to, and enmeshed within, TM likely within the downstream juxtacanalicular portion of the TM where the bulk of outflow resistance is thought to be generated.^52^, ^53^ Provided there is a supply of NO donors, NO can be continuously and locally released and delivered to the outflow resistance sites within the lifetime of the enzymes. A portion of NO donors might be cleared through aqueous outflow; however, segmental flow will tend to cluster β‐gal‐NONOate‐loaded liposomes and β‐galactosidase‐loaded capsules into the active filtration regions of the TM, where their close proximity will minimize the diffusion distance necessary for the substrate to reach the enzyme. By coupling an enzyme‐prodrug therapy mechanism^54^, ^55^, ^56^ with smart biomaterial encapsulation, we localized the delivery of NO to the target tissue, and the desired dose of therapeutics can be tuned by varying the concentration of externally administered NO donors within the liposomes—two highly desirable features for effective NO‐mediated IOP‐lowering therapy.

In conclusion, we have developed a platform for delivering NO to the targeted outflow resistance sites within the conventional outflow pathway, utilizing the strategy of on‐site NO release via enzyme biocatalysis to increase outflow facility. Our platform will allow future manipulation of NO delivery in a dose‐ and time‐dependent manner without concern of off‐target effects, and potentially help compensate for the impaired NO‐regulatory machinery^57^, ^58^, ^59^ within the conventional outflow pathway that contributes toward the pathogenesis of glaucoma. The strategy developed is generic and may open new routes to the next generation of localized therapeutic delivery.

## Experimental Section

*In Vivo Intracameral Injections of β‐galactosidase‐Loaded Capsules*: All animal experiments were conducted in compliance with the Association for Research in Vision and Ophthalmology Statement for the Use of Animals in Ophthalmic and Vision Research under UK Home Office Project License approval for research at Imperial College London (PPL 70/7306). Mice were first anesthetized with ketamine (66.6 mg kg^−1^, Fort Dodge Animal Health) and Domitor (medetomidine hydrochloride, 0.66 mg kg^−1^, Orion Pharma) via intraperitoneal (IP) injection. Each mouse received dilation drops (2.5% w/v phenylephrine hydrochloride and 1% w/v tropicamide, Bausch & Lomb) to both eyes to minimize potential damage to the iris during injection. This was followed by a subcutaneous injection of enrofloxacin antimicrobial (5 mg kg^−1^, Bayer Healthcare). Eyes were kept moist with artificial tears (Vidisic, Bausch & Lomb) prior to intracameral injections. For intracameral injections, mice were secured in place with the assistance of a head holder (model 923‐B, Kopf Instruments) and placed on a warm‐water place mat. Eyes were first cannulated with a pulled glass microneedle (100 µm tip) positioned parallel to the iris and above the limbus, to remove a portion of the aqueous humor in the anterior chamber by capillary action. Intracameral injections were then carried out with a separate pulled glass microneedle filled with 1.5 µL of β‐galactosidase‐loaded PMA capsules (10^6^ capsules µL^−1^, treated eyes) or empty PMA capsules (10^6^ capsules µL^−1^, control eyes) in UltraPure DNase/RNAse‐free dH_2_O (Invitrogen) connected to a 10 µL glass gastight syringe (Hamilton). The needle was inserted into the same cannulation site with the assistance of a micromanipulator, and 1.5 × 10^6^ PMA capsules were delivered to the anterior chamber of the eye. Topical antibiotic ointment (1% w/w Fucithalmic, LEO Pharma) was then applied to the eyes followed by slow removal of the needle from the eye to reduce reflux. After the procedures, the mice were given Antisedan (atipamezole hydrochloride, 1.5 mg kg^−1^, Orion Pharma) via IP injections and placed in a heated chamber (maintained at 28 °C) to allow for faster recovery from anesthesia. The mice were allowed to recover for 48 h post‐intracameral injections, before they were euthanized by overdose of pentobarbital (100 µL, Euthatal, Merial Animal Health) and the eyes were enucleated for ex vivo perfusions.

*Ex Vivo Delivery of β‐gal‐NONOate‐Loaded Liposomes via Mouse Eye Perfusions*: Eyes from mice (post‐intracameral delivery of PMA capsules) were enucleated within 10 min of death by overdose of pentobarbital and stored in phosphate buffered saline (PBS) at room temperature until perfusion, typically within 15 min. Experiments used paired contralateral eyes, which were perfused simultaneously on two identical iPerfusion systems^50^ under identical experimental conditions. Our cannulation method follows previously described techniques.^5^, ^6^, ^60^ Briefly, the eye was affixed to a support using cyanoacrylate glue and submerged in PBS in a thermoregulated bath at 35 °C. A 33‐gauge beveled needle (Nanofil, World Precision Instruments) was used to cannulate the eye, with the tip of the needle positioned in the anterior chamber using a micromanipulator. The pressure and flow rate inside the eye were measured using a wet–wet differential pressure transducer (PX409, Omegadyne) and a thermal flow sensor (SLG64‐0075, Sensirion), respectively. The enucleated eyes were perfused with β‐gal‐NONOate‐loaded liposomes in DBG (50 × 10^−6^ m β‐gal‐NONOate, 10^6^ liposomes). Both treated and control eyes were pressurized from a reservoir at 8 mmHg for 45 min to allow the eyes to acclimatize to the pressure and temperature environment. During this acclimatization period, the anterior chamber was filled with liposomes which flowed toward the filtration‐active regions of the TM, where the β‐galactosidase‐loaded PMA capsules were enmeshed. After the acclimatization period, eyes were perfused over seven increasing pressure steps, from 6 to 15 mmHg with a motorized reservoir.

*Outflow Facility Analysis*: Conventional outflow facility was defined as previously described by Sherwood et al.^50^ Briefly, for each eye, the last 4 min of steady state pressure and flow data for each step were averaged, and a power law regression was fit to the data according to *Q* = *C*_r_(*P/P*_r_)*^β^ P*, where *Q* is the flow rate, *P* is the pressure, *P*_r_ is the reference pressure, *C*_r_ is the reference facility at *P*_r_, and β characterizes the non‐linearity of the data. This model is used rather than the more common linear model, as it was shown that neither the assumption of pressure‐independent facility nor of a finite flow rate at zero pressure are valid in enucleated mouse eyes.^50^ We therefore consider the facility at a “physiological” pressure drop across the outflow pathway in vivo, IOP‐EVP = *P*_r_ = 8 mmHg, and *C*_r_ acts as an indicator of the physiological facility. It should be noted that although *C*_r_ represents the total outflow facility, which comprises trabecular facility and any pressure dependent components of unconventional outflow, the latter are relatively diminutive and hence *C*_r_ is a good representation of the conventional outflow facility. Additionally, based upon population studies, the distribution of outflow facility data is more accurately represented by a lognormal distribution; therefore, outflow facility values are expressed as *C^x^*/ME_95_, where ME_95_ is the 95% margin of error, such that the 95% confidence intervals can be calculated as [C/ME_95_, C×ME_95_]. In order to determine whether the observed treatment effect was statistically significant, a weighted paired *t*‐test was used on the log‐transformed *C*_r_ values according to Sherwood et al.^50^

Detailed methods are available in the Supporting Information.

## Supporting information

As a service to our authors and readers, this journal provides supporting information supplied by the authors. Such materials are peer reviewed and may be re‐organized for online delivery, but are not copy‐edited or typeset. Technical support issues arising from supporting information (other than missing files) should be addressed to the authors.

Supplementary
