# Supplementary material for: Localized and Controlled Delivery of Nitric Oxide to the Conventional Outflow Pathway via Enzyme Biocatalysis: Toward Therapy for Glaucoma
Source: Adv Mater. 2017 Feb 21;29(16):1604932. doi: 10.1002/adma.201604932 (PMC5400071; doi:10.1002/adma.201604932)
Supplement: Supplementary file 1 — Supplementary [file ADMA-29-na-s001.pdf]

Copyright WILEY-VCH Verlag GmbH & Co. KGaA, 69469 Weinheim, Germany, 2017.

ADVANCED MATERIALS

Supporting Information

for *Adv. Mater.*, DOI: 10.1002/adma.201604932

Localized and Controlled Delivery of Nitric Oxide to the Conventional Outflow Pathway via Enzyme Biocatalysis: Toward Therapy for Glaucoma

Rona Chandrawati, Jason Y. H. Chang, Ester Reina-Torres, Coline Jumeaux, Joseph M. Sherwood, W. Daniel Stamer, Alexander N. Zelikin, Darryl R. Overby,* and Molly M. Stevens**

Copyright WILEY-VCH Verlag GmbH & Co. KGaA, 69469 Weinheim, Germany, 2013.

Supporting Information

Localized and Controlled Delivery of Nitric Oxide to the Conventional Outflow Pathway via Enzyme Biocatalysis: Towards Therapy for Glaucoma

Rona Chandrawati, Jason Y. H. Chang, Ester Reina-Torres, Coline Jumeaux, Joseph M. Sherwood, W. Daniel Stamer, Alexander N. Zelikin, Darryl R. Overby,* and Molly M. Stevens**

Dr. R. Chandrawati,[+] [++] C. Jumeaux, Prof. M. M. Stevens
Department of Materials, Department of Bioengineering, and Institute of Biomedical Engineering
Imperial College London
London SW7 2AZ, UK
E-mail: m.stevens@imperial.ac.uk

Dr. J. Y. H. Chang,[+] Dr. E. Reina-Torres, Dr. J. M. Sherwood, Dr. D. R. Overby
Department of Bioengineering
Imperial College London
London SW7 2AZ, UK
E-mail: d.overby@imperial.ac.uk

Prof. W. D. Stamer
Department of Ophthalmology
Duke University School of Medicine
Durham, NC 27710, USA

Dr. A. N. Zelikin
Department of Chemistry and iNANO Interdisciplinary Nanoscience Center
Aarhus University
Aarhus C 8000, Denmark
E-mail: zelikin@chem.au.dk

[+] R. C. and J. Y. H. C. contributed equally to this work
[++] Present address: School of Chemical and Biomolecular Engineering, The University of Sydney, Sydney, NSW 2006, Australia

Synthesis of Thiol-Functionalized Poly(methacrylic acid) (PMA$_{SH}$): PMA with 14 mol% of thiol modification was synthesized through functionalization of the polymer with pyridine dithioethylamine (PDA). A 300 mg of 30 wt% PMA solution (Mw 15000 Da, Polysciences) was charged with 64.6 mg of 1-ethyl-3-(3-dimethylaminopropyl)carbodiimide hydrochloride (EDC, 20 mg mL^{-1} in 10 mM phosphate buffer pH 7.2, Thermo Scientific) and the mixture was stirred for 15 min. Subsequently, 44.5 mg of PDA (20 mg mL^{-1} in 10 mM phosphate buffer pH 7.2, Nanocs) was added to the mixture and the reaction was allowed to proceed overnight. The reaction mixture was purified via extensive dialysis against water and the polymer was isolated via freeze-drying to obtain white powder of PMA$_{PD}$. The degree of thiol functionalization of PMA was characterized by measuring the absorbance of released 2-pyridinethione (λ_{max}= 343 nm) using dithiothreitol (DTT, Sigma-Aldrich) and then derived by correlation with a calibration curve of PDA. The reduced form, PMA$_{SH}$, was prepared by incubating PMA$_{PD}$ in a solution of 0.5 M DTT (100 mg mL^{-1}) in 20 mM 3-(*N*-morpholino)propanesulfonic acid (MOPS) buffer pH 8.0 for at least 15 min at 37 °C followed by dilution with 20 mM sodium acetate (NaOAc) buffer pH 4.0 to a final concentration of 2 mg mL^{-1}.

***Preparation of Alexa Fluor 488-Labeled β-Galactosidase*:** A solution of 10 mg mL^{-1} β-galactosidase (5 mg) in 100 mM carbonate buffer pH 8.3 was incubated with 100 µL of Alexa Fluor 488 succinimidyl ester (1 mg mL^{-1} in DMSO, Molecular Probes). The reaction was allowed to proceed overnight and excess Alexa Fluor 488 was removed through purification by centrifugal filter devices.

***Assembly of β-Galactosidase-Loaded Capsules*:** Amine-functionalized 1 µm-diameter SiO_2 particles (5 wt% solution, microParticles GmbH) were washed with three centrifugation/redispersion cycles (1200 *g*, 30 s) with 10 mM Tris-EDTA buffer pH 7.5.

β-Galactosidase (1 mg mL^{-1} in 10 mM Tris-EDTA buffer pH 7.5, Sigma-Aldrich) was added to the particle solution and adsorption was allowed to proceed for 1 h with constant shaking. The resulting enzyme-coated particles were washed twice with 10 mM Tris-EDTA buffer pH 7.5 and twice with 20 mM NaOAc buffer pH 4.0. Assembly of polymer multilayers was achieved by alternately incubating the particles with thiol-functionalized poly(methacrylic acid) (PMA_{SH}, 1 mg mL^{-1}, 15 min) and poly(*N*-vinylpyrrolidone) (PVP, Mw 10000 Da, 1 mg mL^{-1}, 15 min, Sigma-Aldrich) in NaOAc buffer with constant shaking. The particles were washed with three centrifugation/redispersion cycles (1200 *g*, 30 s) with NaOAc buffer between layers and the process was repeated until four bilayers of PMA_{SH}/ PVP were assembled. The thiols within the polymer layers were crosslinked with 2,2'-dithiodipyridine (0.5 mg mL^{-1}, 15 h, Sigma-Aldrich) in NaOAc buffer. Hollow capsules containing β-galactosidase were obtained by dissolving the SiO_2 cores using a 2 M hydrofluoric acid (HF)/8 M ammonium fluoride (NH_4F) solution for 2 min, followed by multiple centrifugation/washing cycles (4500 *g*, 3 min). *Caution! HF and NH_4F are highly toxic. Extreme care should be taken when handling HF and NH_4F solutions and only small quantities should be prepared.*

Formation of β-Gal-NONOate-Loaded Liposomes: 1-Palmitoyl-2-oleoyl-*sn*-glycero-3-phosphocholine (POPC, 5 mg, Avanti Polar Lipids) was dissolved in chloroform and dried by evaporation of the chloroform under nitrogen for 1 h. The dry lipid film was hydrated with 1 mL of β-gal-NONOate (25 – 75 μM in DBG solution, Cayman Chemical). β-Gal-NONOate-loaded liposomes were obtained by repeated extrusion (31 times) of the lipid solution through 1 μm polycarbonate membranes (Whatman) using a mini-extruder (Avanti Polar Lipids). Non-encapsulated β-gal-NONOate was removed from the liposome suspension by centrifugal

filter devices. Encapsulation efficiency of NONOate-loaded liposomes was reported to be ca. 35%.[1]

Release of Nitric Oxide by β-Galactosidase/β-Gal-NONOate Reactions: NO release kinetics from non-encapsulated and encapsulated β-galactosidase/β-gal-NONOate was compared and determined with a NO-sensitive electrode (ISO-NO Mark II, World Precision Instruments). The NO electrode was first calibrated following the manufacturer's instruction manual. Briefly, the NO probe was placed in a 0.1 M H_2SO_4/ 0.1 M KI solution to polarize the sensor and to obtain a current baseline. NO calibration curve was obtained by measuring changes in current in response to incremental volumes of 50 µM $NaNO_2$ added to the H_2SO_4/KI solution within a glass vial. Conversion of $NaNO_2$ to NO occurred rapidly and followed a 1:1 stoichiometry, such that NO concentration was dictated by the added concentration of $NaNO_2$. To determine NO release kinetics via β-galactosidase catalytic reactions, the NO probe was first equilibrated in a 0.1 mg mL^{-1} β-galactosidase in DBG solution for 2 h at 37 °C. Incremental volumes of 50 µM β-gal-NONOate in DBG solution were added into the β-galactosidase solution and changes in current were measured over time. To determine NO release kinetics from β-galactosidase encapsulated in PMA capsules and β-gal-NONOate loaded in liposomes, the NO probe was equilibrated in a suspension of β-galactosidase-loaded capsules in DBG solution (10^6 capsules μL^{-1}) for 2 h at 37 °C. Liposomes containing different concentrations of β-gal-NONOate (25 – 75 µM, 10^6 liposomes μL^{-1}) were added into the capsule solution and changes in current were measured over time. All NO measurements were performed in mock aqueous humor solution (DBG solution, typically used for *ex vivo* perfusions) at 37 ºC on a hot plate with continuous mixing. The number of β-galactosidase-loaded capsules and β-gal-NONOate-loaded liposomes was determined by flow cytometry (BD LSRFortessa) and nanoparticle tracking analysis (NTA NanoSight LM10), respectively.

Animal Husbandry: All experiments were conducted in compliance with the Association for Research in Vision and Ophthalmology Statement for the Use of Animals in Ophthalmic and Vision Research under UK Home Office Project License approval for research at Imperial College London. This study used C57BL/6 male wild-type (WT) mice subjected to *in vivo* intracameral injections followed by *ex vivo* eye perfusions. Mice were 10 – 12 wk of age, housed in individually ventilated cages, fed *ad libitum*, and maintained at 21 °C with a 12:12-h light-dark cycle, with lights on from 7 AM to 7 PM.

[1] D. J. Suchyta, M. H. Schoenfisch, *Mol. Pharm.* **2015**, *12*, 3569.

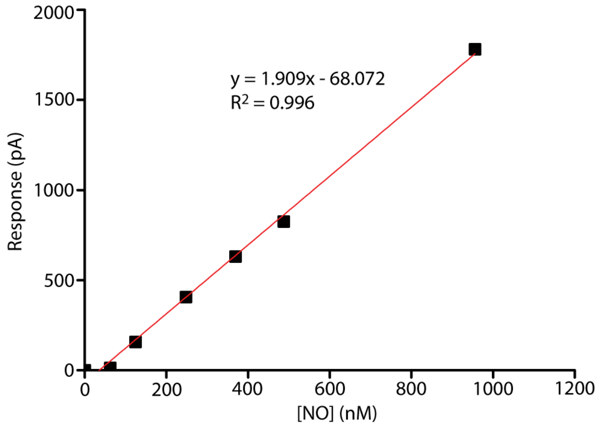

Figure S1. Nitric oxide (NO) calibration curve obtained by measuring changes in current in response to incremental volumes of 50 μM $NaNO_2$ added to a 0.1 M H_2SO_4/0.1 M KI solution.

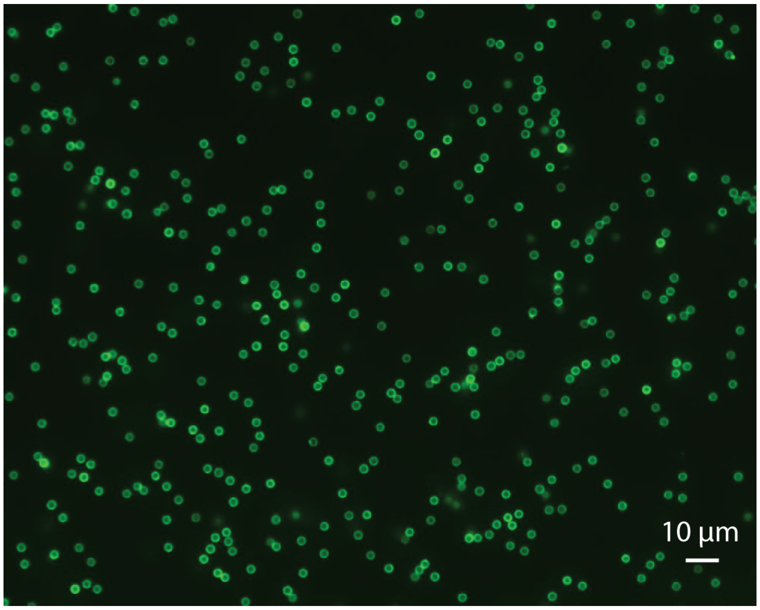

Figure S2. Fluorescence microscopy image of poly(methacrylic acid) capsules containing Alexa Fluor 488-labeled β-galactosidase.
